# Defining Unmet Need Following Lenalidomide Refractoriness: Real-World Evidence of Outcomes in Patients With Multiple Myeloma

**DOI:** 10.3389/fonc.2021.703233

**Published:** 2021-07-21

**Authors:** Catherine S. Y. Lecat, Jessica B. Taube, William Wilson, Jonathan Carmichael, Christopher Parrish, Gabriel Wallis, Charalampia Kyriakou, Lydia Lee, Shameem Mahmood, Xenofon Papanikolaou, Neil K. Rabin, Jonathan Sive, Ashutosh D. Wechalekar, Kwee Yong, Gordon Cook, Rakesh Popat

**Affiliations:** ^1^ Department of Haematology, University College London Hospitals NHS Foundation Trust, London, United Kingdom; ^2^ Department of Haematology, University College London Cancer Institute, London, United Kingdom; ^3^ Department of Oncology, Cancer Research UK & UCL Cancer Trials Centre, University College London, London, United Kingdom; ^4^ Leeds Cancer Centre, Leeds Teaching Hospitals NHS Trust, Leeds, United Kingdom; ^5^ Department of Oncology, The National Institute for Health Research Leeds In Vitro Diagnostics Co-operative (NIHR Leeds MIC), Leeds, United Kingdom

**Keywords:** multiple myeloma, relapsed myeloma, lenalidomide, real-world data, Revlimid, survival outcomes

## Abstract

**Background:**

The treatment paradigm for multiple myeloma (MM) continues to evolve with the development of novel therapies and the earlier adoption of continuous treatments into the treatment pathway. Lenalidomide-refractory patients now represent a challenge with inferior progression free survival (PFS) reported to subsequent treatments. We therefore sought to describe the natural history of MM patients following lenalidomide in the real world.

**Methods:**

This was a retrospective cohort review of patients with relapsed MM who received lenalidomide-based treatments in the U.K. Data were collected for demographics, subsequent therapies, treatment responses, survival outcomes and clinical trial enrollment.

**Results:**

198 patients received lenalidomide-based treatments at a median of 2 prior lines of therapy at a median of 41 months (range 0.5-210) from diagnosis. 114 patients (72% of 158 evaluable) became refractory to lenalidomide. The overall survival (OS) after lenalidomide failure was 14.7 months having received between 0-6 subsequent lines of therapy. Few deep responses were observed with subsequent treatments and the PFS to each further line was < 7 months. There was a steep reduction in numbers of patients able to receive further treatment, with an associated increase in number of deaths. The OS of patients progressing on lenalidomide who did not enter a clinical trial incorporating novel agents was very poor (8.8 months versus 30 months, p 0.0002), although the trials group were a biologically fitter group.

**Conclusion:**

These data demonstrate the poor outcomes of patients failing lenalidomide-based treatments in the real world, the highlight need for more effective treatments.

## Introduction

Multiple myeloma (MM) is an incurable plasma cell malignancy of the bone marrow, characterized by multiple relapses and eventual development of resistant disease. The duration of treatment response typically reduces with each line of therapy, as does the depth of response. A large retrospective study of European real-world data demonstrated that the proportion of patients able to receive treatment reduces with each subsequent line of treatment, with only 15% of patients reaching 4^th^ line treatment and beyond (1). This is likely due to resistant disease, toxicity burden from repeated therapies and age-related co-morbidities. However, recent novel therapy approvals and the development of more optimal drug combinations have translated into improved clinical outcomes, with an expected increase in number of patients receiving later lines of therapy.

Lenalidomide, an immunomodulatory drug (IMiD), is a key backbone agent in the treatment of MM and commonly used as frontline treatment for both transplant eligible and ineligible patients either in combination with proteasome inhibitors (PI), alkylators and/or CD38 monoclonal antibodies or as a doublet with corticosteroids according to performance status (2, 3). Additionally, it is used as maintenance following autologous stem cell transplant (4, 5). In some countries including the U.K., it continues to be used for relapsed MM (6–9) [**Supplementary Figure 1**, (10–13)].

As lenalidomide is typically continued until disease progression or intolerance, most patients become lenalidomide-refractory. Emerging data from sub-group analysis of clinical trials suggest that the treatment response and progression free survival (PFS) of lenalidomide-refractory patients are inferior to those that are sensitive (14–20). Real-world data from RRMM patients who were refractory to an IMiD also demonstrated poor outcomes [**Supplementary Figure 2**, (21–23)]. This highlights a subgroup of patients who are difficult to treat and the need for novel treatment options.

However, there may be discrepancies between clinical trial and real-world outcomes due to multiple patient-related, disease-related and treatment-related factors present between the two groups (24). The observed PFS in real-world data have been shown to be shorter than those reported in clinical trials (25), although there is a lack of clarity to the subsequent responses to treatments. Real-world data can be helpful in identifying outcomes in unselected patient groups, indeed the Connect MM registry suggested that 40% of patients would have been ineligible for inclusion in most randomized controlled trials (26). Whilst there are limitations in real-world datasets (27), they provide valuable insight into the natural history of patients that would otherwise not be known through individual clinical trials.

We therefore sought to understand the long-term outcomes of patients with relapsed or refractory MM (RRMM) following lenalidomide failure in the real-world setting by characterizing the response and PFS to each subsequent treatment and investigating the impact of access to novel agents through clinical trials.

## Materials and Methods

### Study Design and Patient Selection

This was a retrospective, observational chart review study involving two large U.K. myeloma specialist centers (University College London Hospitals NHS Foundation Trust (UCLH) and Leeds Teaching Hospitals NHS Trust). RRMM patients who had previously received lenalidomide between August 2006 and September 2017 were identified using the hospitals’ electronic health record systems. Patients were required to have at least one response assessment with a lenalidomide-based regimen in order to be included in the study. Baseline demographic details, disease characteristics and relevant laboratory blood results were recorded. International Staging System (ISS) at diagnosis and Eastern Cooperative Oncology Group (ECOG) performance status at the time of lenalidomide use were noted. Lenalidomide-based treatment was defined as T0 and subsequent treatments were labelled as T1, T2, T3 etc. Treatment details were extracted, including clinical trial participation and treatment response based on the International Myeloma Working Group (IMWG) uniform response criteria (28).

The National Health Service Health Research Authority deemed that specific research ethical approval was not required due to the anonymous nature of the data collection (REF 704/60/88/81), and this study complied with information governance regulations at both hospitals.

### Study Objectives

The primary objective was to estimate the duration of PFS at each subsequent line of therapy after lenalidomide-based treatment. Secondary objectives included describing overall response rate [ORR, defined as ≥ partial response (PR)] and response categories, overall survival (OS), and outcomes according to participation in clinical trials.

### Statistical Analysis

Qualitative variables were presented as absolute percentage for each modality and quantitative variables were described in terms of mean, median, range and standard deviation. OS was measured from treatment start until death from any cause. PFS was measured from treatment start until whichever came first of disease progression or death from any cause. Patients with no events were censored at the data-cut off of 1^st^ November 2017. OS and PFS were calculated and presented as Kaplan-Meier curves. Differences in OS curves between groups were evaluated with the log-rank test. Cox regression analysis was used to examine the impact of different variables (univariate and multivariate) on OS post-lenalidomide. A p-value less than 0.05 was considered statistically significant. SPSS Statistics and GraphPad Prism were used to generate figures.

## Results

### Patient Characteristics

198 RRMM patients were identified to have commenced lenalidomide-based treatment between August 2006 and September 2017 and had at least one evaluable response assessment. Of these, 159 were treated in UCLH and 39 in Leeds Teaching Hospitals NHS Trust. Patient demographics are shown in **Table 1**.

**Table 1:** Patient characteristics.

Patient characteristics (n = 198)		
Median age at diagnosis/years (range)		60 (33-86)
Median age at lenalidomide commencement/years (range)		66 (35-88)
Median age at progression on lenalidomide/years (range)		67 (36-92)
		**Frequency (%)**
ISS at diagnosis	I	42 (21)
	II	46 (23)
	III	32 (16)
	Unknown	78 (40)
Cytogenetics at diagnosis (High risk defined as t(4;14), del(17/17p), t(14;16), t(14;20), gain(1q))	High risk	28 (14)
	Standard risk	80 (40)
	Unknown	90 (45)
Isotype	IgG	83 (42)
	IgA	34 (17)
	IgD	1 (0.5)
	Light chain	38 (19)
	Non-secretory	2 (1)
	Unknown	40 (20)
Treatment line at which lenalidomide was commenced	2nd	36 (18)
	3rd	110 (56)
	4th	52 (26)
Prior PI/thalidomide exposure	Prior PI and thalidomide	146 (74)
	PI only	49 (25)
	Thalidomide only	2 (1)
	Neither	1 (0.5)

### Outcomes With Lenalidomide and Overall Survival

The median age at the start of lenalidomide based therapy (T0) was 66 years (range 35-88). Patients received a median of 2 prior treatment lines before T0 (18%: 1 prior line, 82%: 2-3 prior lines). The majority of patients (n=146, 74%) received both a PI and thalidomide prior to lenalidomide therapy. Patients commenced lenalidomide at a median of 41 months from diagnosis (range 0.5-210), predominantly as doublet lenalidomide-dexamethasone regimen (n=138, 86% of 159 evaluable). Other regimens used include bortezomib-lenalidomide-dexamethasone (n=6, 4%), ixazomib-lenalidomide-dexamethasone (n=6, 4%), lenalidomide-dexamethasone-elotuzumab (n=2, 1.5%), daratumumab-lenalidomide-dexamethasone (n=5, 3%), cyclophosphamide-lenalidomide-dexamethasone (n=1, 0.6%), and lenalidomide-conditioned reduced intensity allogeneic stem cell transplant (n=1, 0.6%). Lenalidomide doses at disease progression were available in 98 patients (**Supplementary Figure 3A**).

**Figure 1:**
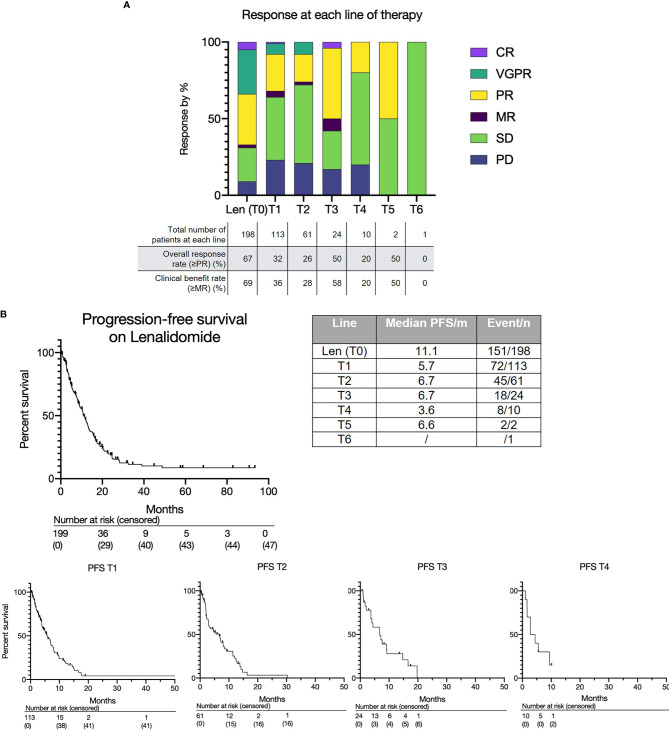
**(A)** Treatment response to lenalidomide-based treatment and subsequent lines of therapy. **(B)** Progression free survival for lenalidomide-based therapy (T0) and each subsequent line (T1, T2, T3 etc.).

**Figure 2:**
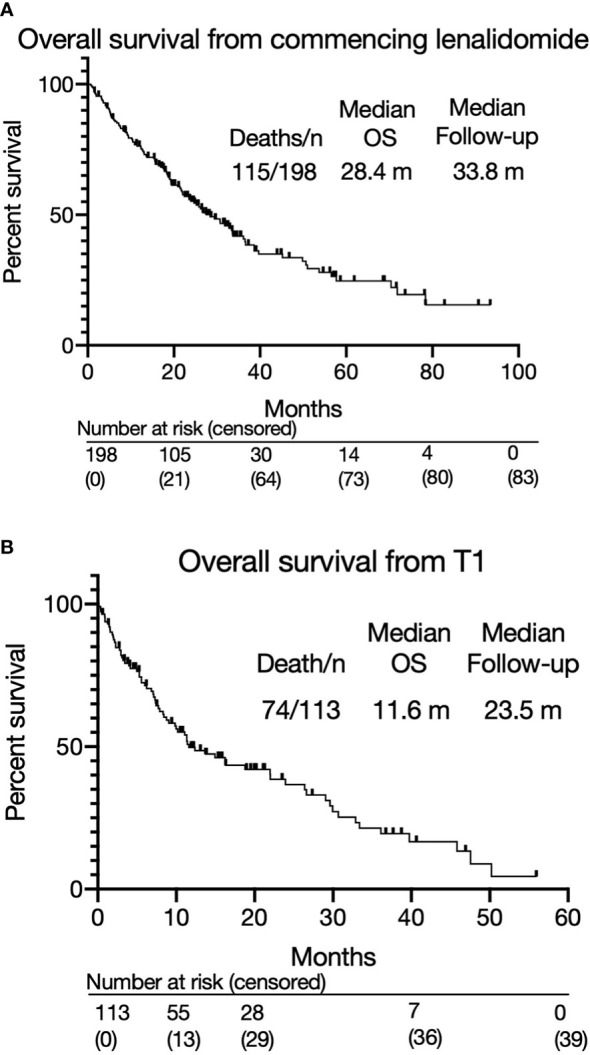
**(A)** Overall survival from commencing lenalidomide-based therapy. **(B)** Overall survival from T1.

The overall response rate to lenalidomide-based treatment was 67% (n=133) (**Figure 1A**): PR, 66 (33%); very good partial response (VGPR), 58 (29%); complete response (CR), 9(5%). The median PFS from T0 was 11.1 months (range 0.2-93.4) (**Figure 1B**) and median OS was 28.4 months with a median follow up of 33.8 months (**Figure 2A**). The median OS from IMWG defined disease progression on lenalidomide or change of therapy for another reason was 14.7 months. The median OS from T1 was 11.6 months as shown in **Figure 2B**. Those who achieved a response to lenalidomide had a superior OS than those who did not (38.6 months with VGPR/PR versus 12.3 months with MR/SD/PD, p<0.0001, see **Supplementary Figure 6A**). Those who became refractory to lenalidomide also had a shorter OS than those who did not (median OS 26.2 months *versus* not reached, p<0.0001, see **Supplementary Figure 6B**). In an exploratory analysis of the sub-group that had doses of lenalidomide recorded, there was no significant difference in PFS2 or OS for those progressing on lenalidomide 25mg *vs* <25mg (PFS2: 16 months *vs* 28.4 months respectively p=0.24; OS: 36.7months *vs* 22.0 months respectively p=0.055) (**Supplementary Figure 3B**).

As of data cut-off, 41 patients had not progressed on lenalidomide, of whom 31 were still alive and 10 had died. Lenalidomide was stopped due to toxicity in 11 (7%) patients. The majority of patients (n=112, 71% of 158 evaluable) became refractory to lenalidomide after an initial response. Despite this, 81 (51%) continued on lenalidomide for a median of 4.14 months (range 0.1-31.5) after evidence of progressive disease (PD). Out of these 81 patients, 24 continued on lenalidomide for more than 6 months. Overall, 31 (15.7% of 198 total population) patients progressed on lenalidomide and died without receiving any further treatment.

### Subsequent Lines of Therapy Post-Lenalidomide

**Figure 3:**
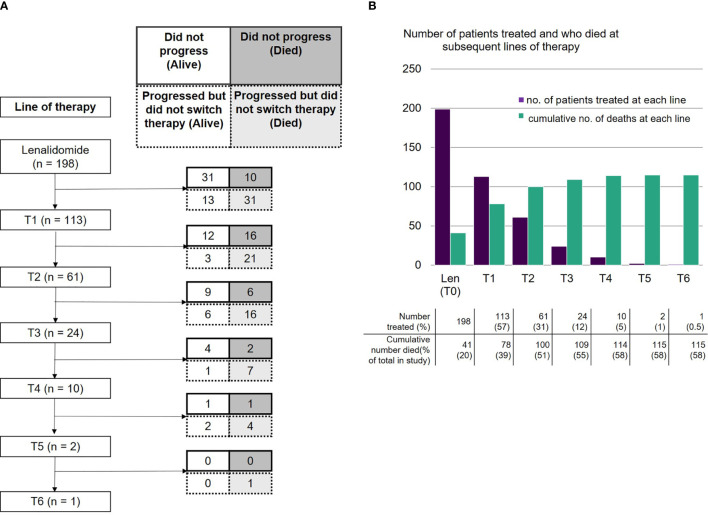
**(A)** Number of patients receiving lenalidomide-based therapy and each subsequent line, including those who did not progress (alive or died) and those who progressed but did not switch therapy (alive or died). **(B)** Number of patients who were treated with lenalidomide-based therapy and each subsequent line, and cumulative number of deaths at each line.

The absolute numbers of patients that were able to receive treatment diminished at each subsequent line after lenalidomide (**Figure 3A**): 113 patients (57%) received the next line of treatment after lenalidomide (T1), 61 (31%) received a further line (T2), 24 (12%) reached the subsequent line (T3) and 10 (5%) reached T4. Only two patients remained on treatment at T5 and beyond. The drop in patients able to receive subsequent lines of treatment was predominantly due to deaths during that line of treatment (**Figure 3B**). A smaller number had either PD but not yet changed treatment, or had not yet progressed. Approximately a third of subjects died at each line from T1 to T3.

A variety of other treatments were used immediately after lenalidomide. The most common was a pomalidomide containing regimen, although some were enrolled in clinical trials, or received alternative treatments including low dose palliative chemotherapy (for full list, see **Supplementary Figure 4**).

The overall and depth of response was limited at sequential lines of treatment (**Figure 1A** and **Supplementary Figure 5**). Overall response rates (≥PR) were as follows: T1 - 32% (36/113); T2 - 26% (16/61); T3 - 50% (12/24); T4 - 20% (2/10); T5 - 50% (1/2); T6 - 0% (0/1). Most patients achieved at least stable disease; deeper responses (≥VGPR) were rarely observed. The median PFS for each subsequent treatment was also short at 5.7 months at T1, 6.6 months at T2, 6.7 months at T3 and 3.6 months at T4 (**Figure 1B**).

**Figure 4:**
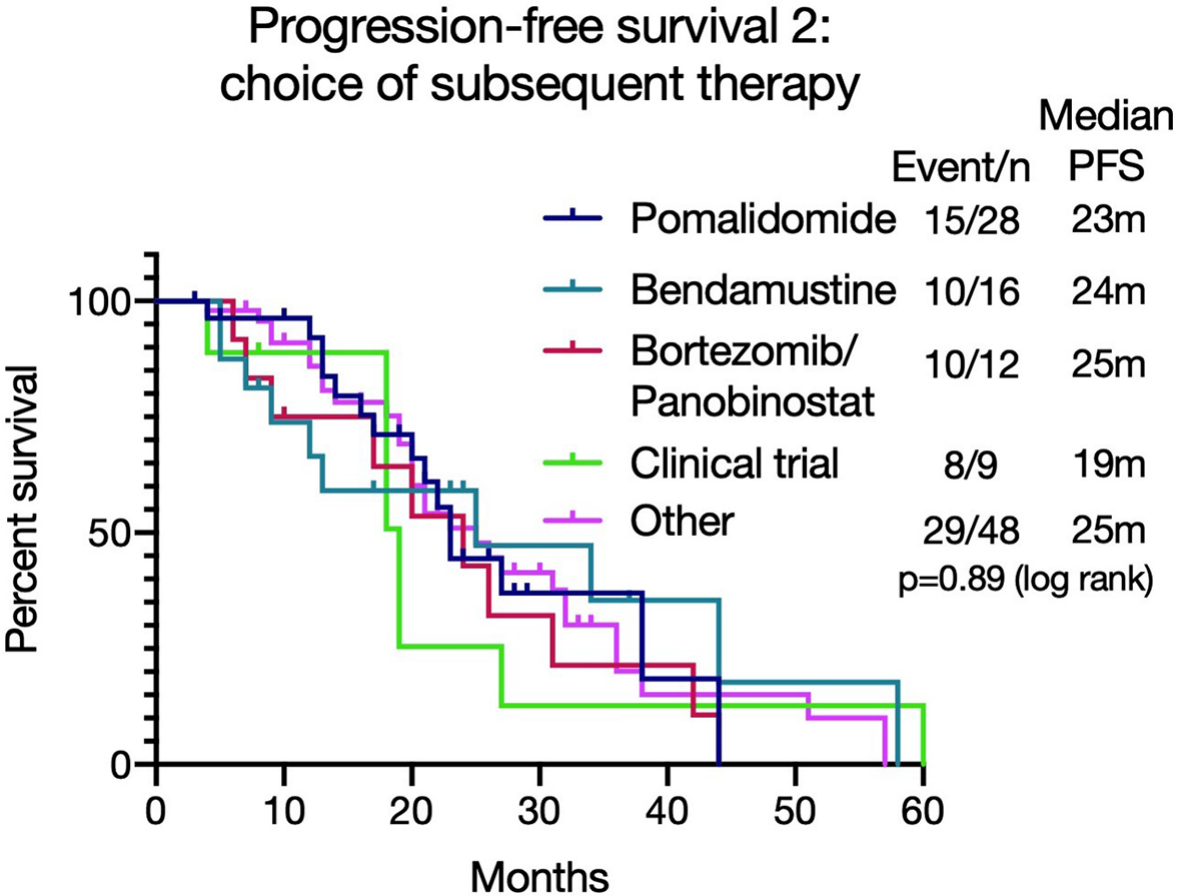
Progression free survival 2 [PFS2 - from commencing lenalidomide to progression on next line of therapy (T1)] based on different treatment choices after lenalidomide-based therapy. The median PFS2 was similar irrespective of treatment choice (p=0.89).

As the majority (112/158, 71%) of patients were refractory to lenalidomide at the beginning of the next treatment (T1), PFS2 [from commencing lenalidomide to progression on next line of therapy (T1)] was assessed to review if there was an optimal salvage treatment for such patients, taking into consideration the duration of response to lenalidomide. The median PFS2 was similar irrespective of treatment choice (pomalidomide (n=28): 23 months, bortezomib and Panobinostat (n=12): 24 months, bendamustine (n=16): 25 months, with clinical trials (n=9): 19 months, other therapies (n=48): 25 months (p=0.89 (log rank)) (**Figure 4**). For those who received pomalidomide at T1, the PFS was significantly longer in those who achieved a longer (over 6 months) PFS with lenalidomide (7.04 months versus 2.78 months, p=0.038, see **Supplementary Figure 7**).

### Clinical Trial Participation and Overall Survival

**Figure 5:**
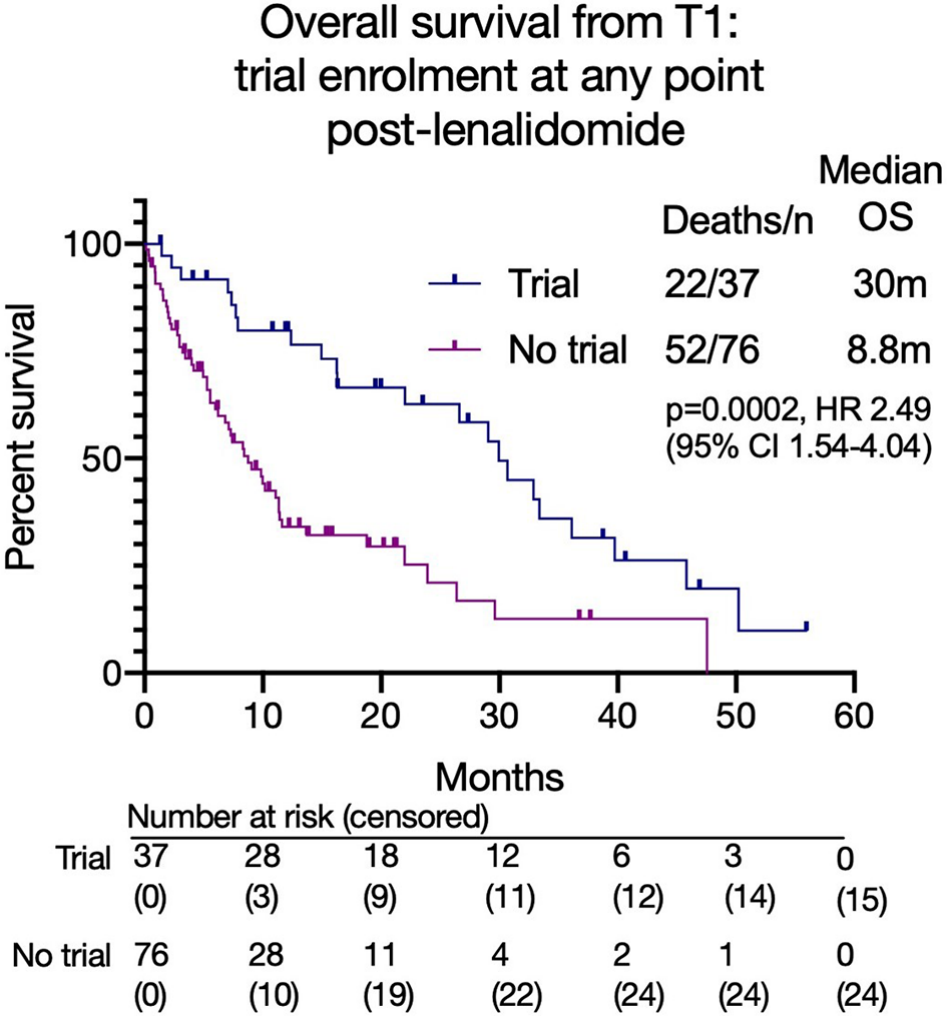
Overall survival from T1 based on clinical trial enrolment at any time point after lenalidomide-based treatment.

**Figure 6:**
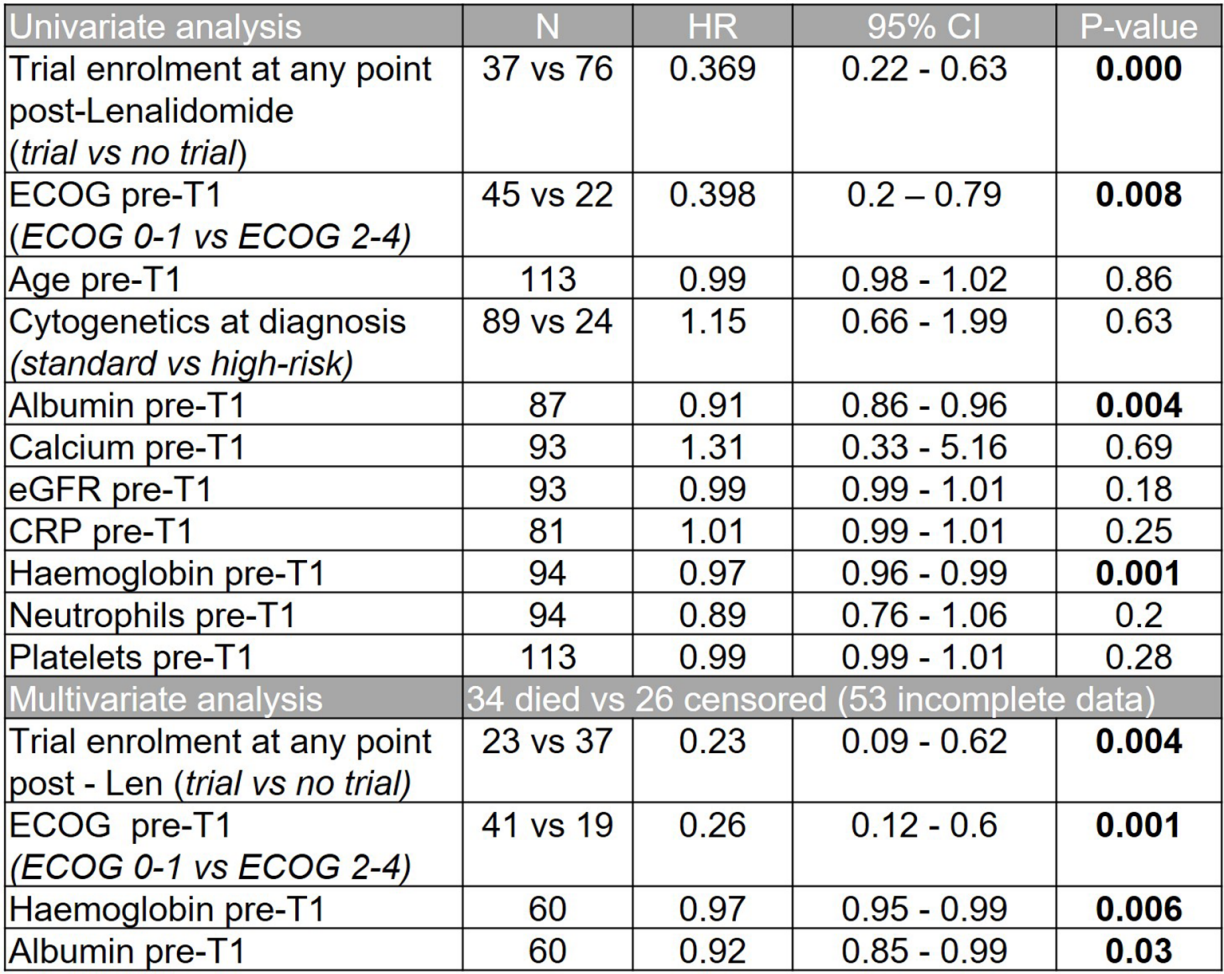
Univariate and multivariate analyses showing impact of patient variables on overall survival from T1.

Overall, 37 patients (33%) enrolled in a clinical trial at any time after lenalidomide-based treatment. These patients had a superior median OS from T1 to those that did not (30.0 months versus 8.8 months, p=0.0002; HR 2.41, 95% CI 1.53 - 3.80, **Figure 5**). However, a high early mortality was noted in the non-trial group with a 6-month mortality of 91.9% (non-trial) versus 63.2% (trial) (p=0.0017, HR 3.2, 95% CI 1.5-6.7) from commencing T1. In univariate analysis of OS, C-reactive protein (CRP), platelet or neutrophil count, estimated glomerular filtration rate (eGFR, MDRD), high risk cytogenetics or patient age had no impact. However, subsequent trial enrollment, good performance status (ECOG 0-1), higher hemoglobin and higher albumin were all associated with significantly better overall survival. Significance was maintained in a multivariable model of these 4 variables, however some were excluded from this model as they did not have complete data (**Figure 6**).

## Discussion

The current treatment paradigm for MM involves continuous treatment until disease progression. Therefore, patients become refractory to treatments, which subsequently limit further options. Many patients become refractory to lenalidomide early on in their treatment pathway and this group have inferior outcomes compared to those who are not, as demonstrated in published studies as well as our dataset. Additionally, during the current COVID-19 pandemic some patients would have deferred ASCT and are continuing on lenalidomide instead. Understanding the natural history of patients following lenalidomide in the real world can therefore advise optimal management and help design future trials.

This study demonstrated that the survival after failing lenalidomide at 3^rd^ line for relapsed MM was poor at 14.7 months, with fewer patients able to receive subsequent lines. Due to limited available data, a difference in outcomes for those progressing on full treatment dose lenalidomide versus a lower dose was not noted. This remains an area of interest. Subsequent response rates following lenalidomide were low with very few deep responses (≥VGPR) observed. Additionally, the median PFS for each subsequent line was less than 7 months and more patients died at each subsequent line. These observations suggest that patients should be treated with optimal treatment as early as possible, rather than reserving treatments for later lines, as not all patients will live to reach this point. As treatment advances continue, new and effective treatments are under evaluation. The advent of B-cell maturation antigen (BCMA) targeted treatments such as Belantamab Mafodotin, chimeric antigen reception T-cells (CAR-T) and T-cell engagers as well as the incorporation of antibodies with standard to care regimens will lead to more effective salvage regimens for relapsed patients. Indeed, the ORR and durations of response to these treatments are already demonstrating improvements over historical data (29). However, these agents are not all yet routinely available and when they are licensed, they may be restricted in some countries. Therefore, it was of interest that patients enrolled into clinical trials had an improved survival to those that did not. This may be in part due to the effectiveness of the novel treatments; however, the early mortality of the non-trial patients as well as the difference in parameters such as hemoglobin and albumin suggests that the clinical trial group was potentially a biologically fitter group which is not surprising given the selection criteria for trials. Nevertheless, this data supports enrollment of patients into clinical trials to access novel treatments, although the impact could be greater if eligibility criteria were not so strict.

It is of interest that some patients continued on treatment with lenalidomide for over 6 months after IMWG defined disease progression due to lack of clinical relapse. This is relevant for treatment funders that may assume that treatments are stopped at the time of IMWG defined progression. Indeed, the clonal heterogeneity and patient variability observed in MM requires personalized decisions to be made based on the clinical phenotype of disease, genetic risk and patient preference, and as such some patients with indolent relapses continued on treatment for longer. There was no difference in PFS2 according to immediate next treatment, and so this dataset was unable to recommend an optimal treatment for lenalidomide-refractory patients, although those that had a PFS < 6 months had a shorter PFS with pomalidomide and should be considered for a class switch to a proteasome inhibitor-based combination. Ongoing clinical trials will be critical to guide this.

This study is limited by its retrospective nature, the heterogeneity of the lenalidomide-refractory cohort who received lenalidomide in line 1 to 3, and that newer agents and combination regimens have been approved or made available since the beginning of data collection in 2006. In addition, this data focuses on the use of lenalidomide in the relapsed setting whereas today it can be used at first line. However, this historical data allows longer follow-up and the ability to describe longitudinal outcomes according to each subsequent line. It also provides interesting data describing that natural plight of MM patients that switch from treatment to treatment across multiple lines at relapse.

Of note, one large U.S. real-world multi-sites myeloma dataset where 23.8% of patients were treated with lenalidomide upfront demonstrated a median PFS of 11.5 months (25), similar to our median lenalidomide PFS of 11.1 months. Furthermore, lenalidomide remains a treatment option only in relapsed settings for some countries across the world. Another limitation is that the number of patients in later lines of treatment are small, but this represents the eventual incurable nature of the disease despite multiple lines of treatment. Further research examining quality of life in the real world would be of interest for these patients.

In conclusion, these data provide valuable insights into the real-world outcomes of patients with relapsed refractory MM that have failed lenalidomide and highlights an unmet need for the development of more effective treatment strategies.

## Data Availability Statement

The raw data supporting the conclusions of this article will be made available by the corresponding author upon reasonable request, without undue reservation.

## Author Contributions

CL and JT are joint first authors. CL, JT, KY, GC, and RP designed the study. CP, GW, CK, LL, SM, XP, NR, JS, AW, KY, GC, and RP provided data. CL, JT, and JC collected the data. CL, JT, and WW analyzed the data. CL, JT, and RP wrote the paper. All authors contributed to the article and approved the submitted version.

## Conflict of Interest

The authors declare that the research was conducted in the absence of any commercial or financial relationships that could be construed as a potential conflict of interest.
