# Supplementary material for: Defining Unmet Need Following Lenalidomide Refractoriness: Real-World Evidence of Outcomes in Patients With Multiple Myeloma
Source: Front Oncol. 2021 Jul 21;11:703233. doi: 10.3389/fonc.2021.703233 (PMC8335564; doi:10.3389/fonc.2021.703233)
Supplement: Supplementary file 1 [file Presentation_1.pptx]

## Slide 1
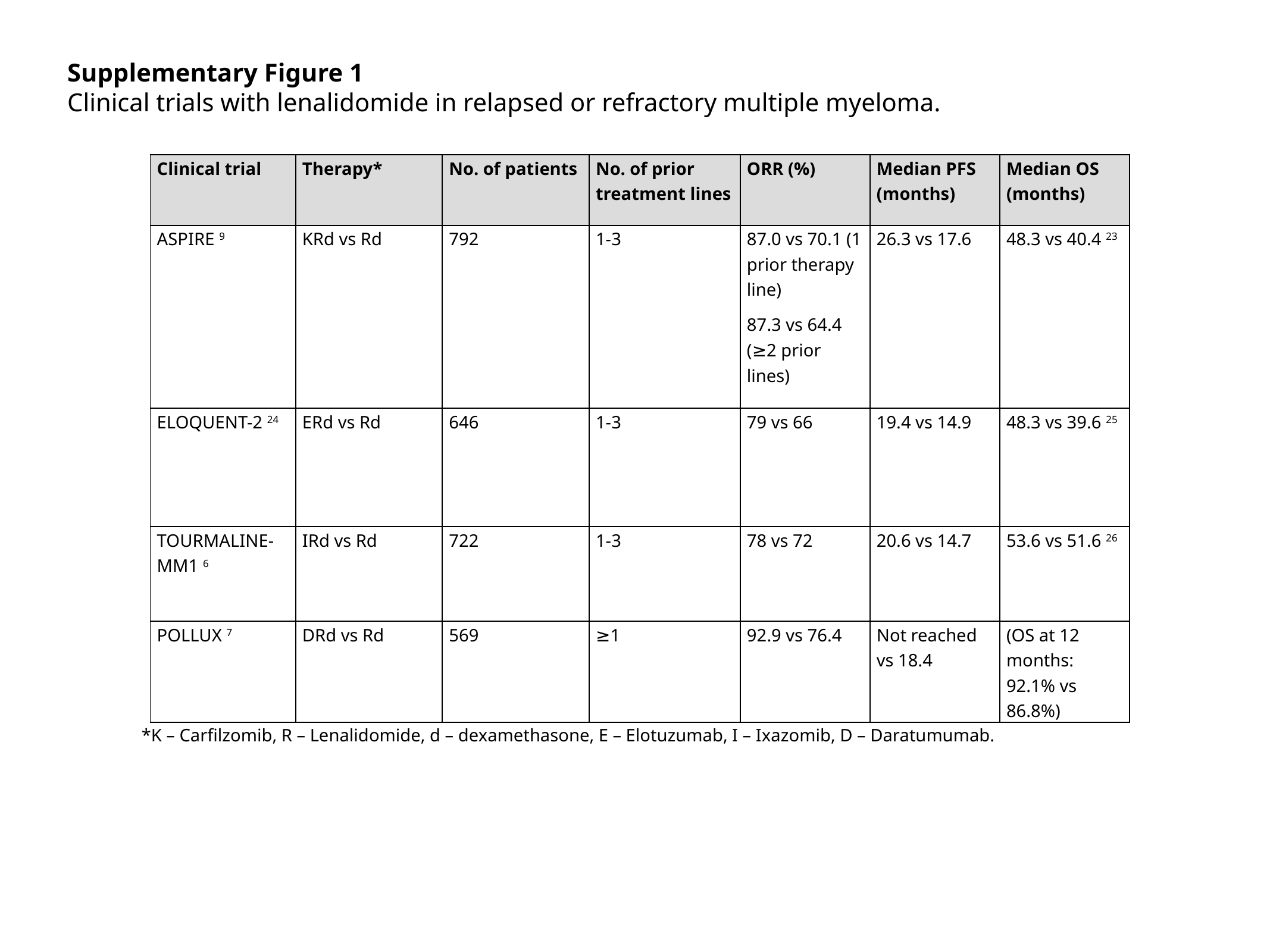

# Supplementary Figure 1 Clinical trials with lenalidomide in relapsed or refractory multiple myeloma.
| Clinical trial | Therapy\* | No. of patients | No. of prior treatment lines | ORR (%) | Median PFS (months) | Median OS (months) |
| --- | --- | --- | --- | --- | --- | --- |
| ASPIRE 9 | KRd vs Rd | 792 | 1-3 | 87.0 vs 70.1 (1 prior therapy line) 87.3 vs 64.4 (≥2 prior lines) | 26.3 vs 17.6 | 48.3 vs 40.4 23 |
| ELOQUENT-2 24 | ERd vs Rd | 646 | 1-3 | 79 vs 66 | 19.4 vs 14.9 | 48.3 vs 39.6 25 |
| TOURMALINE-MM1 6 | IRd vs Rd | 722 | 1-3 | 78 vs 72 | 20.6 vs 14.7 | 53.6 vs 51.6 26 |
| POLLUX 7 | DRd vs Rd | 569 | ≥1 | 92.9 vs 76.4 | Not reached vs 18.4 | (OS at 12 months: 92.1% vs 86.8%) |
*K – Carfilzomib, R – Lenalidomide, d – dexamethasone, E – Elotuzumab, I – Ixazomib, D – Daratumumab.

## Slide 2
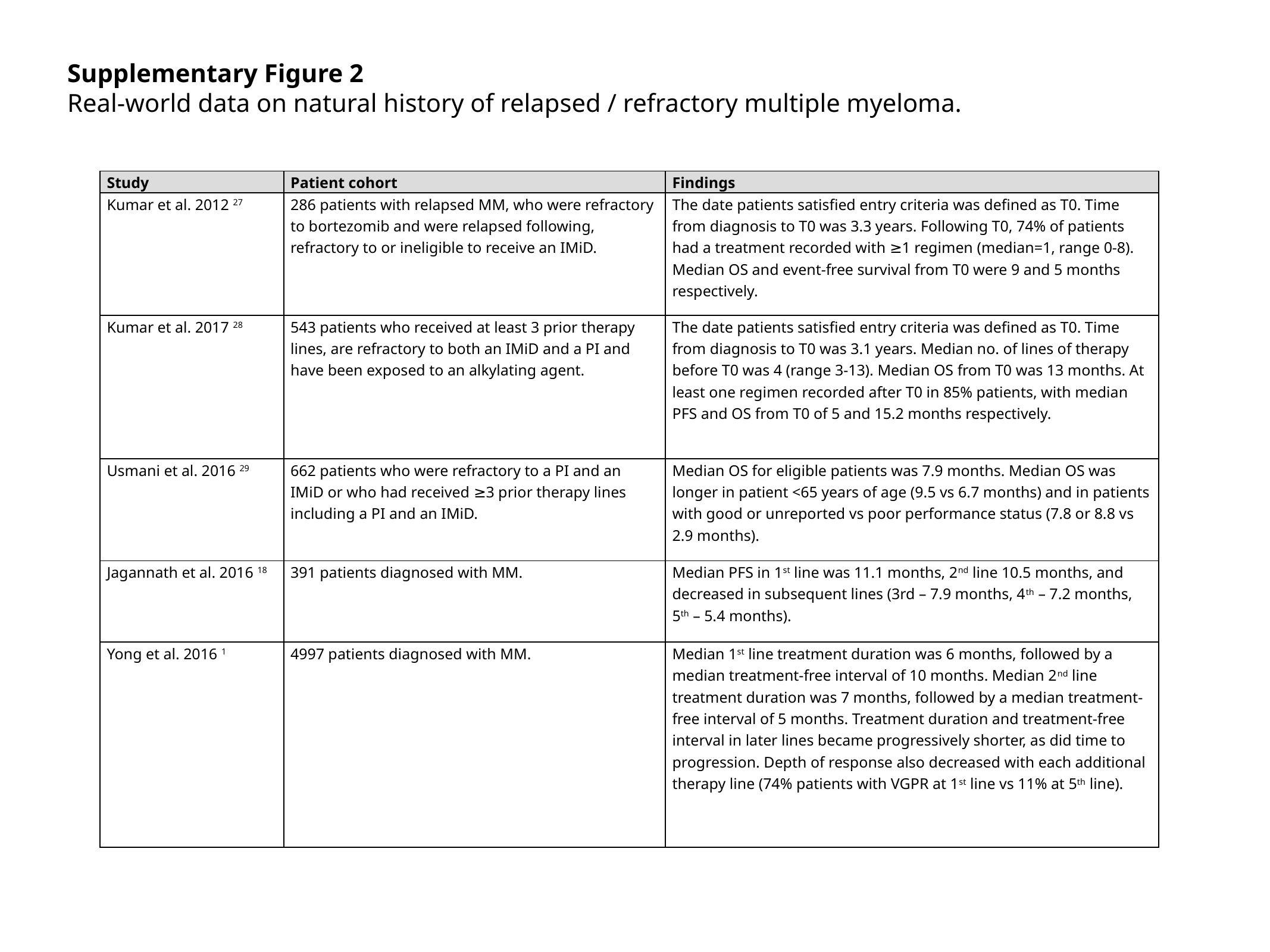

# Supplementary Figure 2 Real-world data on natural history of relapsed / refractory multiple myeloma.
| Study | Patient cohort | Findings |
| --- | --- | --- |
| Kumar et al. 2012 27 | 286 patients with relapsed MM, who were refractory to bortezomib and were relapsed following, refractory to or ineligible to receive an IMiD. | The date patients satisfied entry criteria was defined as T0. Time from diagnosis to T0 was 3.3 years. Following T0, 74% of patients had a treatment recorded with ≥1 regimen (median=1, range 0-8). Median OS and event-free survival from T0 were 9 and 5 months respectively. |
| Kumar et al. 2017 28 | 543 patients who received at least 3 prior therapy lines, are refractory to both an IMiD and a PI and have been exposed to an alkylating agent. | The date patients satisfied entry criteria was defined as T0. Time from diagnosis to T0 was 3.1 years. Median no. of lines of therapy before T0 was 4 (range 3-13). Median OS from T0 was 13 months. At least one regimen recorded after T0 in 85% patients, with median PFS and OS from T0 of 5 and 15.2 months respectively. |
| Usmani et al. 2016 29 | 662 patients who were refractory to a PI and an IMiD or who had received ≥3 prior therapy lines including a PI and an IMiD. | Median OS for eligible patients was 7.9 months. Median OS was longer in patient <65 years of age (9.5 vs 6.7 months) and in patients with good or unreported vs poor performance status (7.8 or 8.8 vs 2.9 months). |
| Jagannath et al. 2016 18 | 391 patients diagnosed with MM. | Median PFS in 1st line was 11.1 months, 2nd line 10.5 months, and decreased in subsequent lines (3rd – 7.9 months, 4th – 7.2 months, 5th – 5.4 months). |
| Yong et al. 2016 1 | 4997 patients diagnosed with MM. | Median 1st line treatment duration was 6 months, followed by a median treatment-free interval of 10 months. Median 2nd line treatment duration was 7 months, followed by a median treatment-free interval of 5 months. Treatment duration and treatment-free interval in later lines became progressively shorter, as did time to progression. Depth of response also decreased with each additional therapy line (74% patients with VGPR at 1st line vs 11% at 5th line). |

## Slide 3
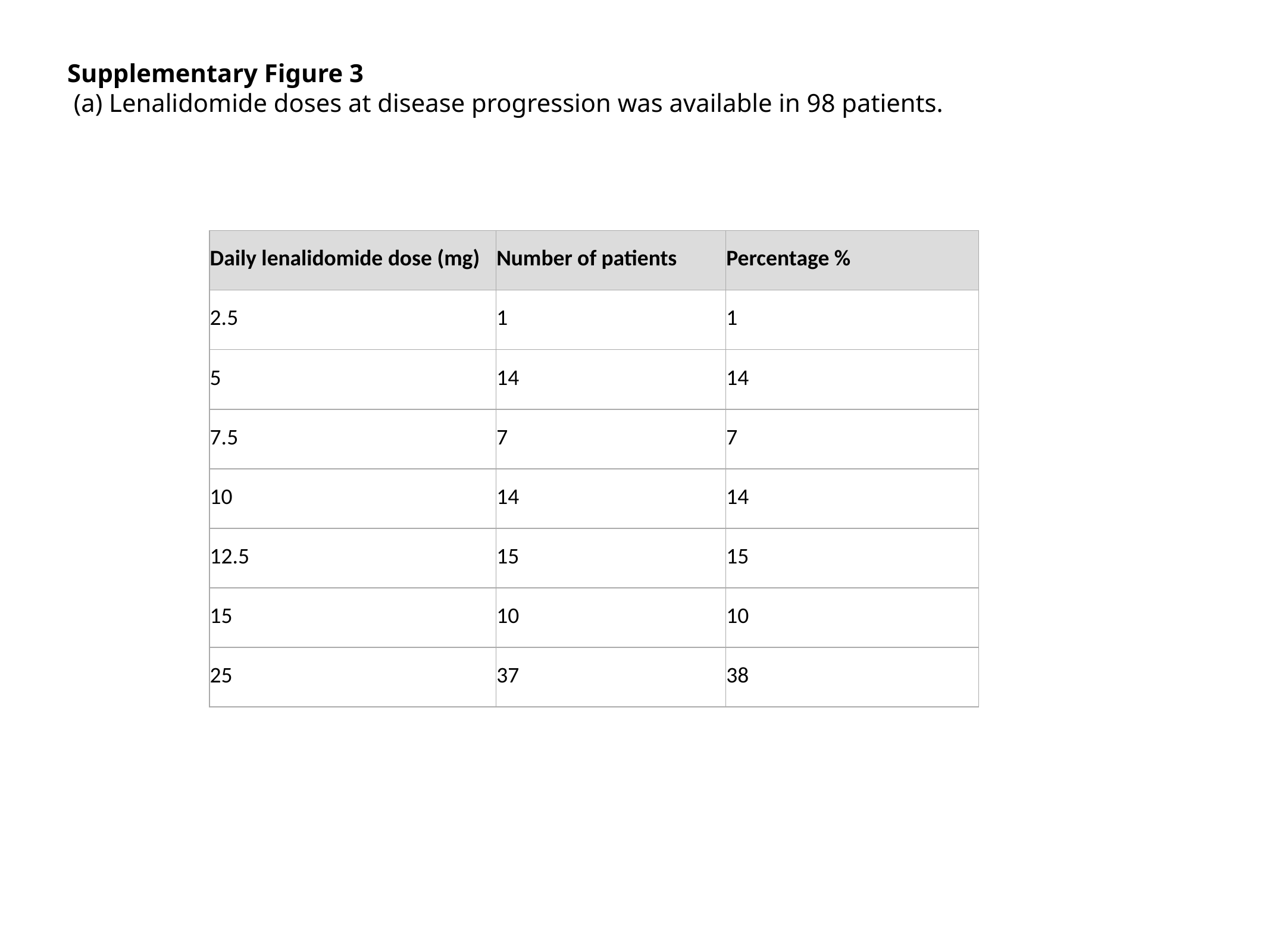

# Supplementary Figure 3 (a) Lenalidomide doses at disease progression was available in 98 patients.
| Daily lenalidomide dose (mg) | Number of patients | Percentage % |
| --- | --- | --- |
| 2.5 | 1 | 1 |
| 5 | 14 | 14 |
| 7.5 | 7 | 7 |
| 10 | 14 | 14 |
| 12.5 | 15 | 15 |
| 15 | 10 | 10 |
| 25 | 37 | 38 |

## Slide 4
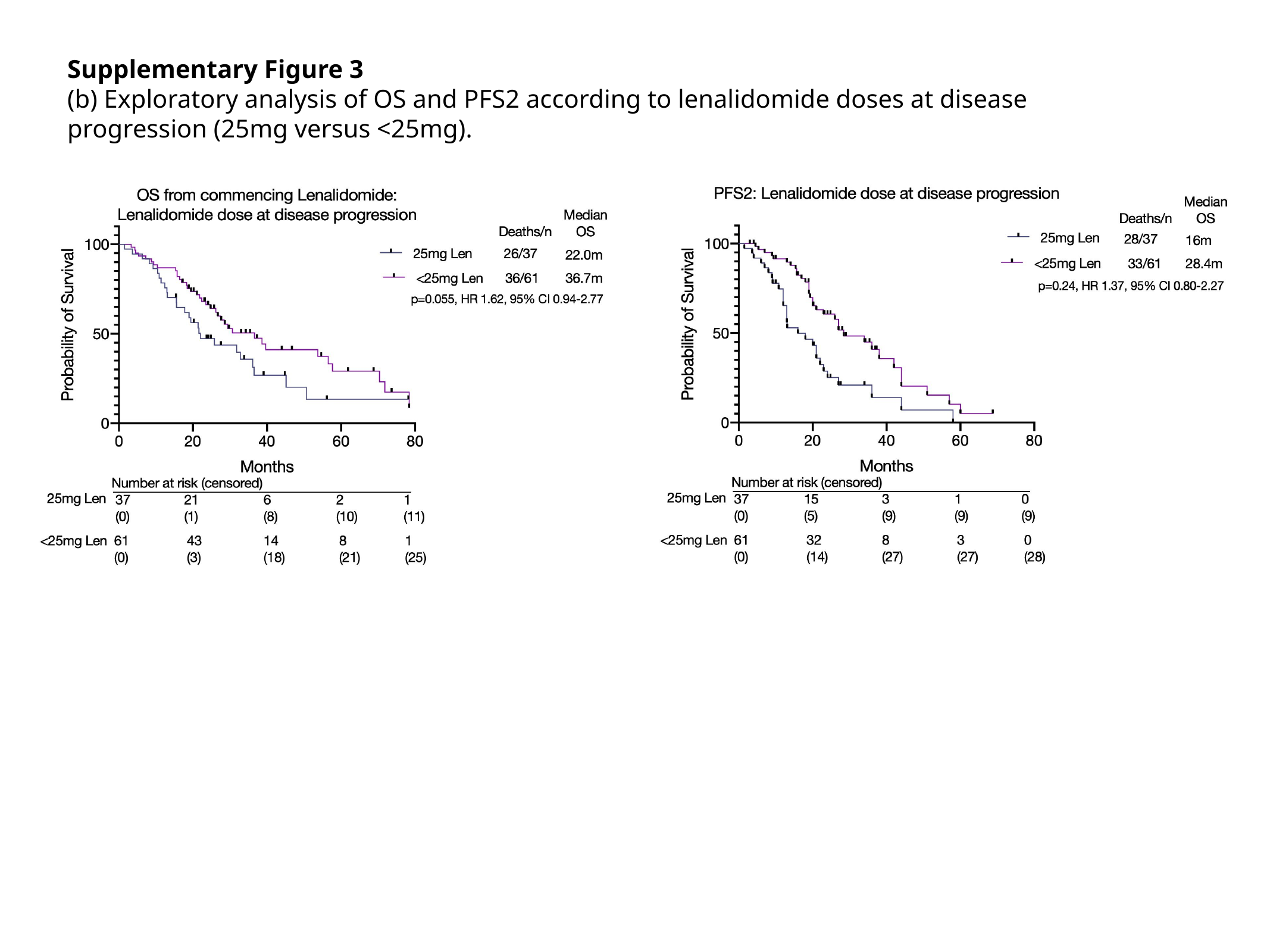

Supplementary Figure 3
(b) Exploratory analysis of OS and PFS2 according to lenalidomide doses at disease progression (25mg versus <25mg).

## Slide 5
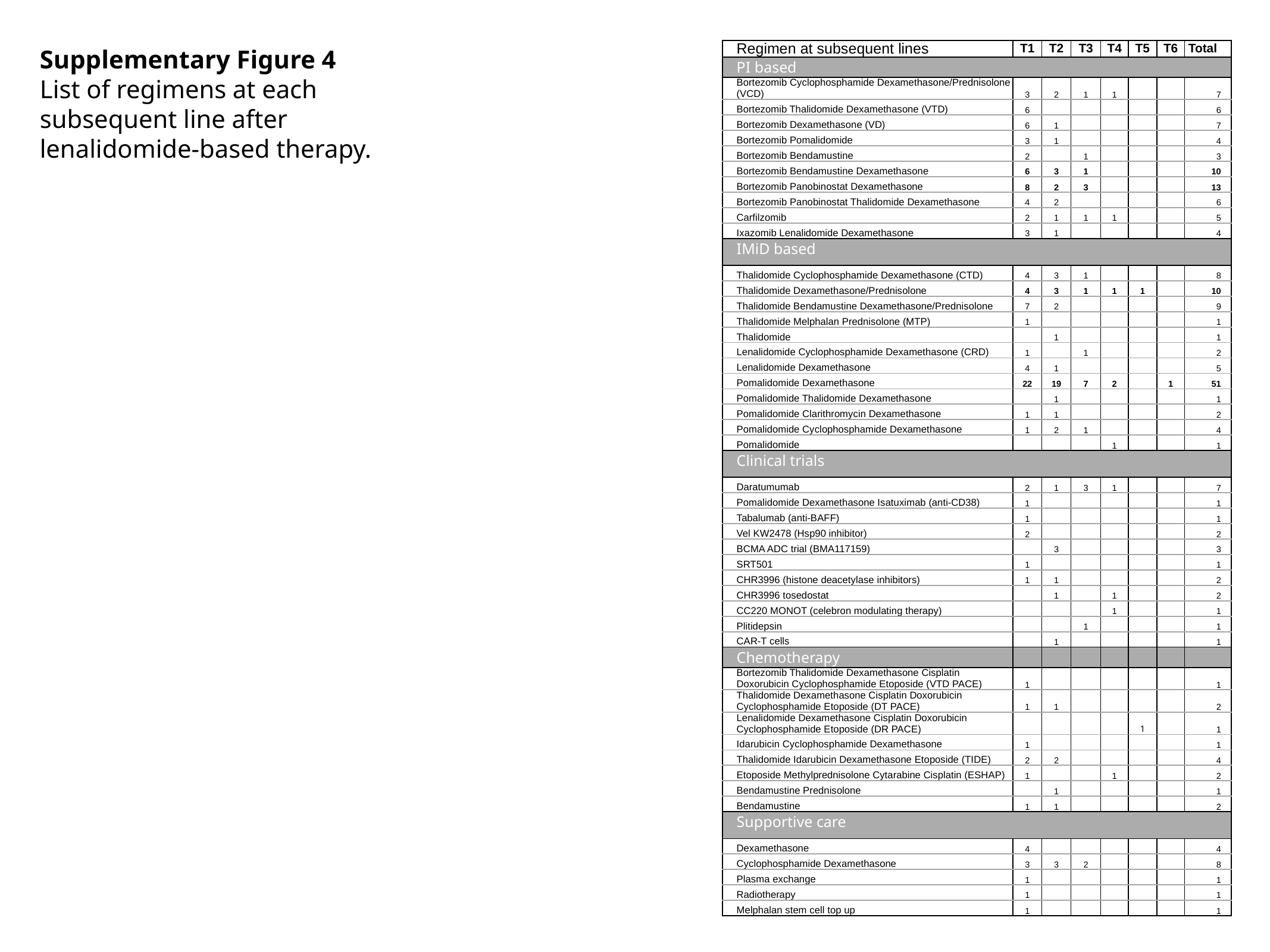

# Supplementary Figure 4 List of regimens at each subsequent line after lenalidomide-based therapy.
| Regimen at subsequent lines | T1 | T2 | T3 | T4 | T5 | T6 | Total |
| --- | --- | --- | --- | --- | --- | --- | --- |
| PI based | | | | | | | |
| Bortezomib Cyclophosphamide Dexamethasone/Prednisolone (VCD) | 3 | 2 | 1 | 1 | | | 7 |
| Bortezomib Thalidomide Dexamethasone (VTD) | 6 | | | | | | 6 |
| Bortezomib Dexamethasone (VD) | 6 | 1 | | | | | 7 |
| Bortezomib Pomalidomide | 3 | 1 | | | | | 4 |
| Bortezomib Bendamustine | 2 | | 1 | | | | 3 |
| Bortezomib Bendamustine Dexamethasone | 6 | 3 | 1 | | | | 10 |
| Bortezomib Panobinostat Dexamethasone | 8 | 2 | 3 | | | | 13 |
| Bortezomib Panobinostat Thalidomide Dexamethasone | 4 | 2 | | | | | 6 |
| Carfilzomib | 2 | 1 | 1 | 1 | | | 5 |
| Ixazomib Lenalidomide Dexamethasone | 3 | 1 | | | | | 4 |
| IMiD based | | | | | | | |
| Thalidomide Cyclophosphamide Dexamethasone (CTD) | 4 | 3 | 1 | | | | 8 |
| Thalidomide Dexamethasone/Prednisolone | 4 | 3 | 1 | 1 | 1 | | 10 |
| Thalidomide Bendamustine Dexamethasone/Prednisolone | 7 | 2 | | | | | 9 |
| Thalidomide Melphalan Prednisolone (MTP) | 1 | | | | | | 1 |
| Thalidomide | | 1 | | | | | 1 |
| Lenalidomide Cyclophosphamide Dexamethasone (CRD) | 1 | | 1 | | | | 2 |
| Lenalidomide Dexamethasone | 4 | 1 | | | | | 5 |
| Pomalidomide Dexamethasone | 22 | 19 | 7 | 2 | | 1 | 51 |
| Pomalidomide Thalidomide Dexamethasone | | 1 | | | | | 1 |
| Pomalidomide Clarithromycin Dexamethasone | 1 | 1 | | | | | 2 |
| Pomalidomide Cyclophosphamide Dexamethasone | 1 | 2 | 1 | | | | 4 |
| Pomalidomide | | | | 1 | | | 1 |
| Clinical trials | | | | | | | |
| Daratumumab | 2 | 1 | 3 | 1 | | | 7 |
| Pomalidomide Dexamethasone Isatuximab (anti-CD38) | 1 | | | | | | 1 |
| Tabalumab (anti-BAFF) | 1 | | | | | | 1 |
| Vel KW2478 (Hsp90 inhibitor) | 2 | | | | | | 2 |
| BCMA ADC trial (BMA117159) | | 3 | | | | | 3 |
| SRT501 | 1 | | | | | | 1 |
| CHR3996 (histone deacetylase inhibitors) | 1 | 1 | | | | | 2 |
| CHR3996 tosedostat | | 1 | | 1 | | | 2 |
| CC220 MONOT (celebron modulating therapy) | | | | 1 | | | 1 |
| Plitidepsin | | | 1 | | | | 1 |
| CAR-T cells | | 1 | | | | | 1 |
| Chemotherapy | | | | | | | |
| Bortezomib Thalidomide Dexamethasone Cisplatin Doxorubicin Cyclophosphamide Etoposide (VTD PACE) | 1 | | | | | | 1 |
| Thalidomide Dexamethasone Cisplatin Doxorubicin Cyclophosphamide Etoposide (DT PACE) | 1 | 1 | | | | | 2 |
| Lenalidomide Dexamethasone Cisplatin Doxorubicin Cyclophosphamide Etoposide (DR PACE) | | | | | 1 | | 1 |
| Idarubicin Cyclophosphamide Dexamethasone | 1 | | | | | | 1 |
| Thalidomide Idarubicin Dexamethasone Etoposide (TIDE) | 2 | 2 | | | | | 4 |
| Etoposide Methylprednisolone Cytarabine Cisplatin (ESHAP) | 1 | | | 1 | | | 2 |
| Bendamustine Prednisolone | | 1 | | | | | 1 |
| Bendamustine | 1 | 1 | | | | | 2 |
| Supportive care | | | | | | | |
| Dexamethasone | 4 | | | | | | 4 |
| Cyclophosphamide Dexamethasone | 3 | 3 | 2 | | | | 8 |
| Plasma exchange | 1 | | | | | | 1 |
| Radiotherapy | 1 | | | | | | 1 |
| Melphalan stem cell top up | 1 | | | | | | 1 |

## Slide 6
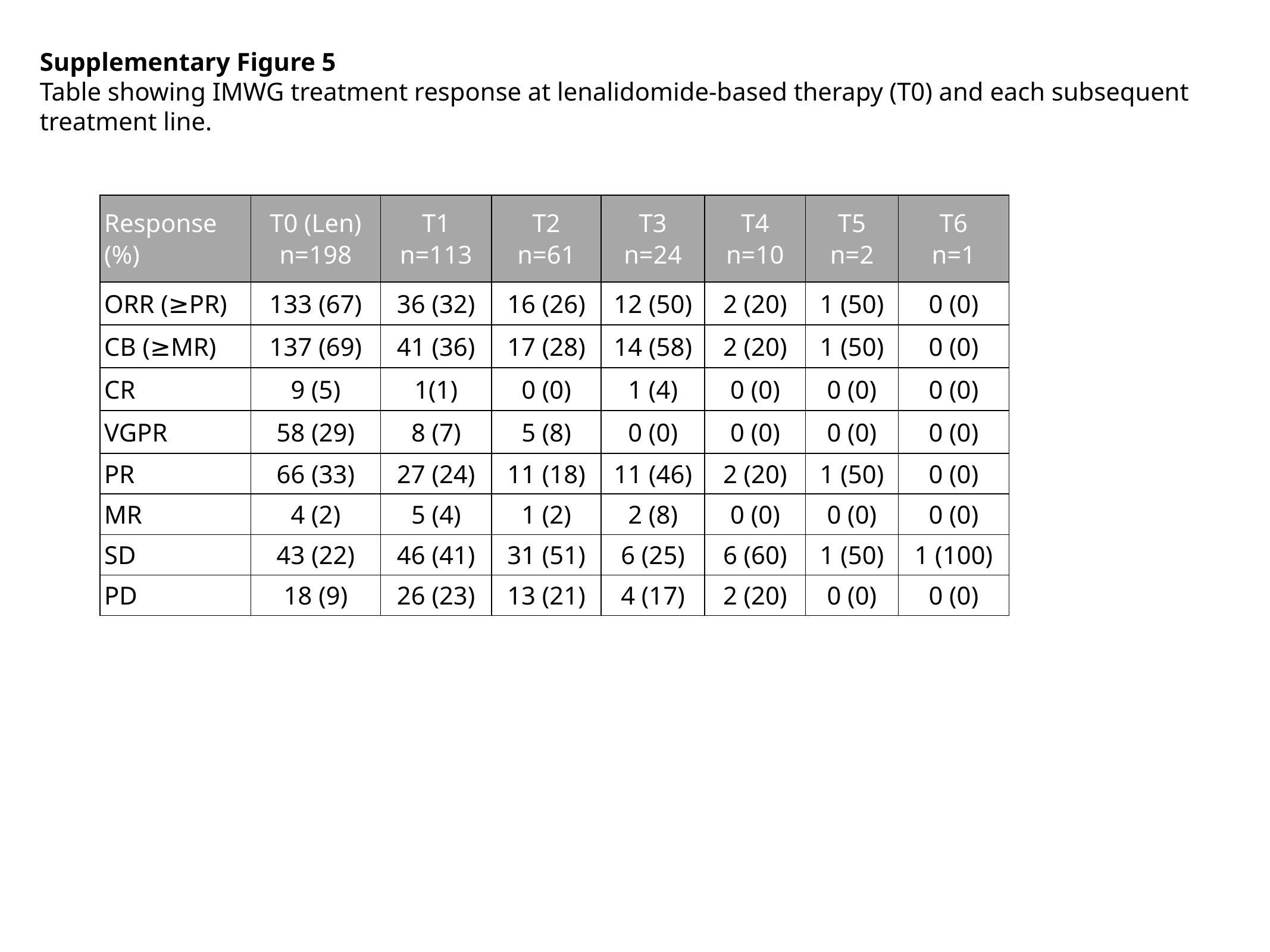

# Supplementary Figure 5 Table showing IMWG treatment response at lenalidomide-based therapy (T0) and each subsequent treatment line.
| Response (%) | T0 (Len) n=198 | T1 n=113 | T2 n=61 | T3 n=24 | T4 n=10 | T5 n=2 | T6 n=1 |
| --- | --- | --- | --- | --- | --- | --- | --- |
| ORR (≥PR) | 133 (67) | 36 (32) | 16 (26) | 12 (50) | 2 (20) | 1 (50) | 0 (0) |
| CB (≥MR) | 137 (69) | 41 (36) | 17 (28) | 14 (58) | 2 (20) | 1 (50) | 0 (0) |
| CR | 9 (5) | 1(1) | 0 (0) | 1 (4) | 0 (0) | 0 (0) | 0 (0) |
| VGPR | 58 (29) | 8 (7) | 5 (8) | 0 (0) | 0 (0) | 0 (0) | 0 (0) |
| PR | 66 (33) | 27 (24) | 11 (18) | 11 (46) | 2 (20) | 1 (50) | 0 (0) |
| MR | 4 (2) | 5 (4) | 1 (2) | 2 (8) | 0 (0) | 0 (0) | 0 (0) |
| SD | 43 (22) | 46 (41) | 31 (51) | 6 (25) | 6 (60) | 1 (50) | 1 (100) |
| PD | 18 (9) | 26 (23) | 13 (21) | 4 (17) | 2 (20) | 0 (0) | 0 (0) |

## Slide 7
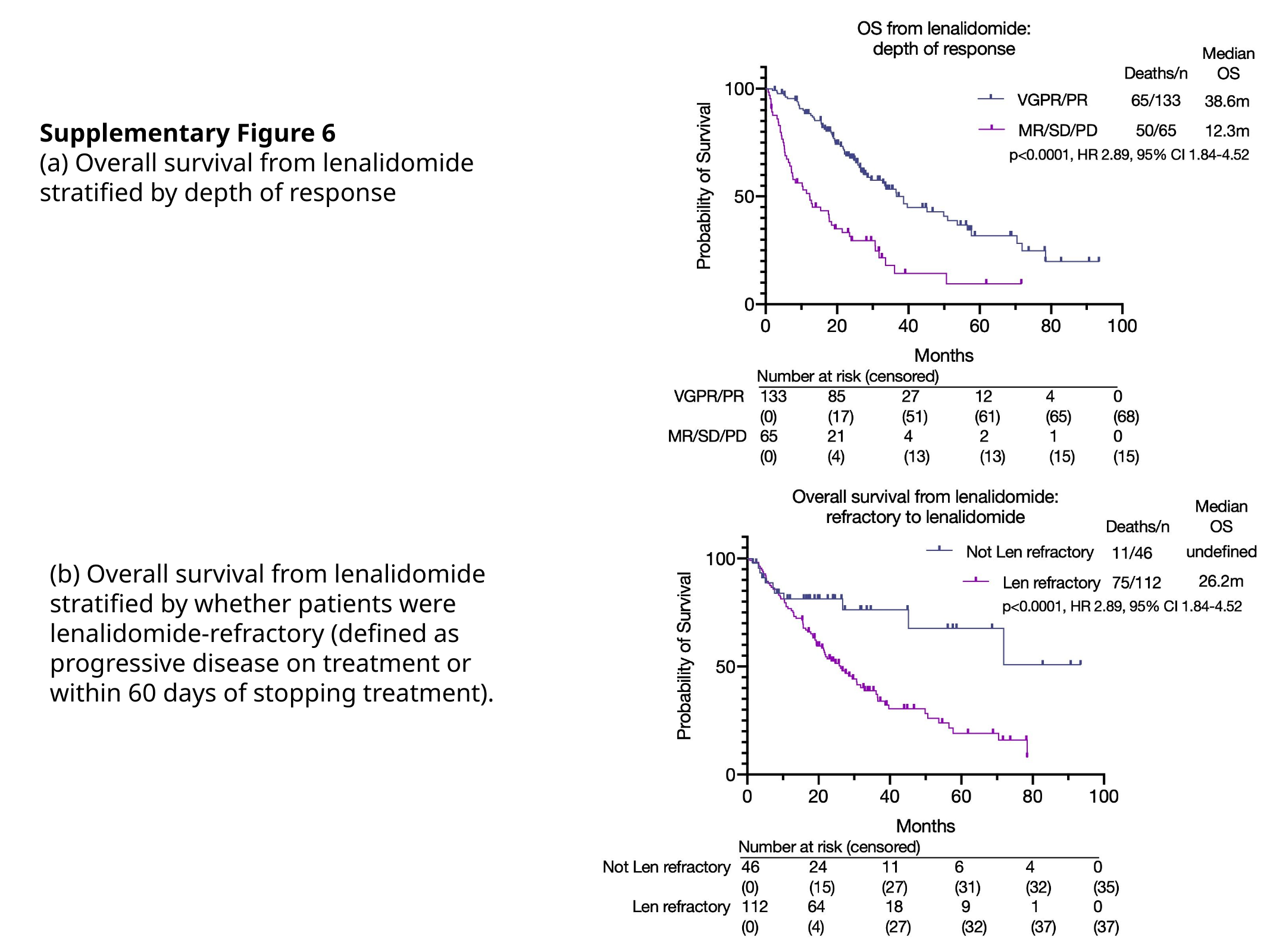

Supplementary Figure 6
(a) Overall survival from lenalidomide stratified by depth of response
(b) Overall survival from lenalidomide stratified by whether patients were lenalidomide-refractory (defined as progressive disease on treatment or within 60 days of stopping treatment).

## Slide 8
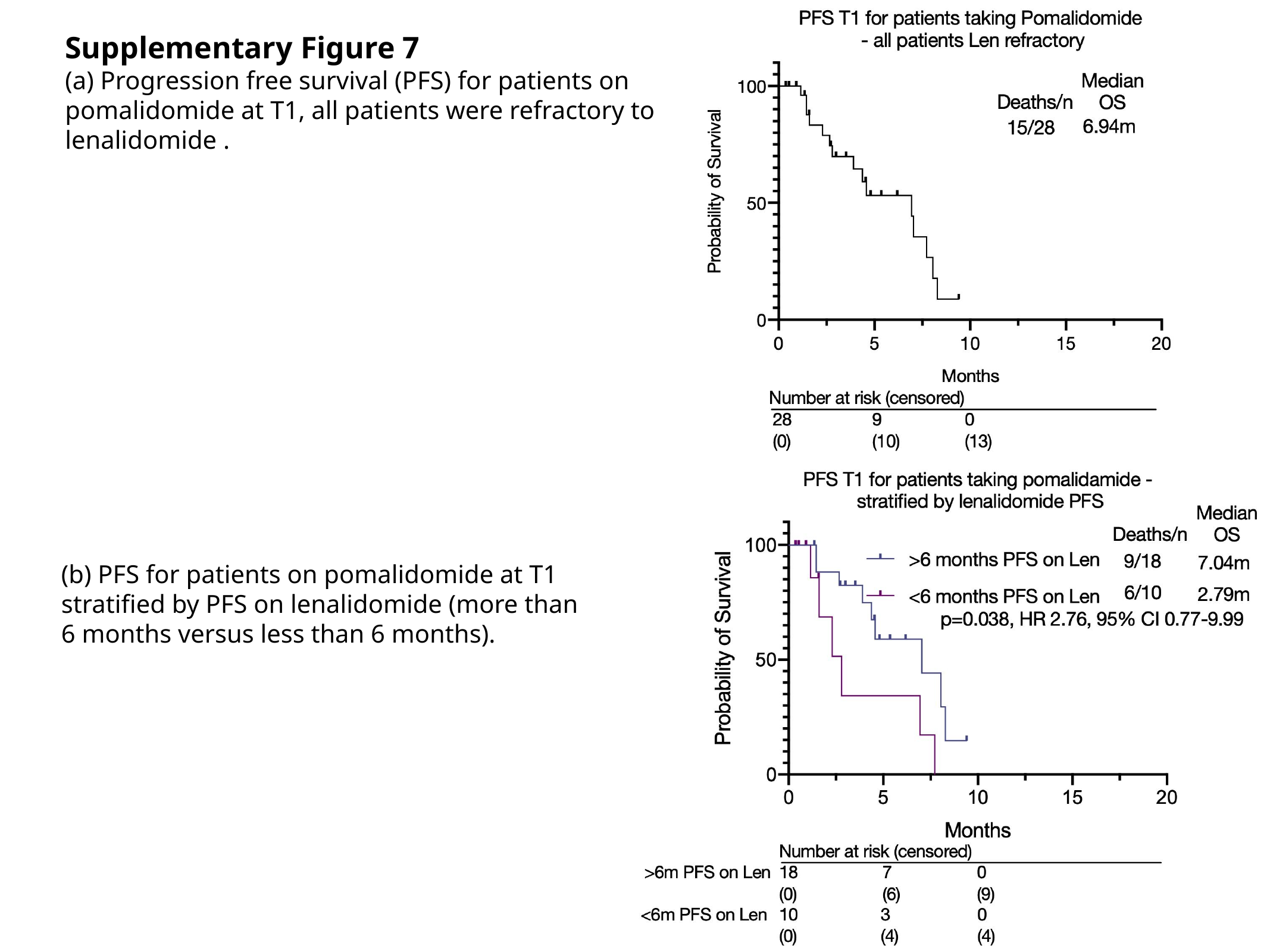

Supplementary Figure 7
(a) Progression free survival (PFS) for patients on pomalidomide at T1, all patients were refractory to lenalidomide .
(b) PFS for patients on pomalidomide at T1 stratified by PFS on lenalidomide (more than 6 months versus less than 6 months).
